# Green production of a yellow laccase by *Coriolopsis gallica* for phenolic pollutants removal

**DOI:** 10.1186/s13568-022-01434-6

**Published:** 2022-07-16

**Authors:** Qingjing Cen, Xiaodan Wu, Leipeng Cao, Yanjuan Lu, Xuan Lu, Jianwen Chen, Guiming Fu, Yuhuan Liu, Roger Ruan

**Affiliations:** 1 State Key Laboratory of Food Science and Technology, Engineering Research Center for Biomass Conversion of Ministry of Education, Nanchang University, Nanchang, 330047 Jiangxi China; 2 International Institute of Food Innovation, Nanchang University, Nanchang, 330047 Jiangxi China; 3 Beijing Fairyland Environmental Technology CO., LTD, Beijing, 100096 China; 4 Department of Bioproducts and Biosystems Engineering, Center for Biorefining, University of Minnesota, St. Paul, MN 55108 USA

**Keywords:** Yellow laccase, *Coriolopsis gallica*, Agricultural wastes, Enzymatic properties, Phenolic pollutants

## Abstract

As a group of green biocatalysts, fungal laccases have aroused great interest in diverse biotechnological fields. Therein, yellow laccase has advantages over blue laccase in catalytic performance, but it is not common in the reported fungal laccases. Here, we report a yellow laccase from white-rot fungus *Coriolopsis gallica* NCULAC F1 about its production, purification, characterization, and application. Laccase production in the co-fermentation of pomelo peel and wheat bran reached the enzyme activity by 10,690 U/L after 5 days with a 13.58-time increase. After three steps of purification, laccase increased the specific activity from 30.78 to 188.79 U/mg protein with an activity recovery of 45.64%. The purified *C. gallica* laccase (CGLac) showed a molecular mass of about 57 kDa. CGLac had a yellow color and no absorption peaks at 610 nm and 330 nm, suggesting that it’s a yellow laccase. CGLac exhibited stability towards temperature (40–60 °C) and neutral pH (6.0–8.0). Fe^3+^ and Mn^2+^ strongly stimulated CGLac activity by 162.56% and 226.05%, respectively. CGLac remained high activities when exposed to organic reagents and putative inhibitors. Additionally, CGLac contributed to 90.78%, 93.26%, and 99.66% removal of phenol, *p*-chlorophenol and bisphenol A after 120 min, respectively. In conclusion, a green efficient production strategy was introduced for fungal laccase, and the obtained CGLac presented great enzymatic properties and catalytic potential in the removal of phenolic pollutants.

**Supplementary Information:**

The online version contains supplementary material available at 10.1186/s13568-022-01434-6.

## Key points

- Pomelo peels and wheat bran are great nutritional sources and laccase inducers.
- CGLac showed the spectral characteristic of yellow laccase.
- CGLac had great stability and catalytic ability for phenolic pollutants removal.

**Supplementary Information:**

The online version contains supplementary material available at 10.1186/s13568-022-01434-6.

## Introduction

Phenols are an array of widespread contaminants in industrial effluents, mainly being produced in the tanning industry (Yuan et al. 2021), pulp and paper industry (Mandeep et al. 2019), olive oil mill processing industry (Bouknana et al. 2019), and other emissions. Up to now, multiple types of phenolic pollutants (PPs) usually are co-existent and recalcitrant in wastewater, resulting in complex treatment. In recent years, with the serious threat of PPs to the environment and public health, enzyme-mediated strategies for the biodegradation of PPs have attracted more and more attention (Singh et al. 2021). Laccases, as a group of polyphenol oxidases, have raised increasing interest in the biodegradation of PPs because of various advantages of low substrate specificity and efficient oxidation (Arregui et al. 2019). In a recent review (Morsi et al. 2020), laccases also are accentuated as the smart, greener, and futuristic biocatalysts to degrade the emerging pollutants. Altogether, laccase is considered a biocatalytic candidate for PPs biotreatments (biodegradation, bioremediation, detoxification) in wastewater (Bilal et al. 2019; Kumar and Chandra 2021).

Laccases (EC 1.10.3.2, Lac) belong to the copper-containing oxidoreductases family and are widely distributed in nature, mostly derived from bacteria, fungi, lichens, higher plants, and insects (Forootanfar and Faramarzi 2015). Therein, fungal laccases have been researched extensively due to their abundant sources, good characteristics, and excellent ability to oxidize various organic compounds (Baldrian 2006). Typical fungal laccases have three copper sites and belong to “blue” laccases (Rivera-Hoyos et al. 2013). Unlike typical laccases, which have been extensively studied, some unusual laccases, such as “yellow” laccase and “white” laccase, can oxidize pertinacious compounds in the absence of mediators and are therefore gaining increasing attention in biotechnology applications (Ademakinwa and Agboola 2016; Ike et al. 2019). Although atypical laccases have been well characterized with high catalytic capacity and a wide substrate range, a few studies focused on their production processes. With the increasing demand for laccase catalysts, enough yield of laccase has become the prerequisite for the development of laccase employment on a large industrial scale. At present, how to produce high-quality laccase effectively is still the main task.

Induction is effective to achieve high laccase production since the expression level of laccase can be improved by inducers (Alessandra Piscitelli et al. 2011; Debnath and Saha 2020). Although aromatic mediators and metal ions were widely used as inducers to increase the laccase yield (Zhuo et al. 2017), the negative effects of these inducers on the organisms and environment shouldn’t be neglected due to their toxicity and pollution risk (Wang et al. 2019). Therefore, the utilization of some natural inducers for the enhancement of laccase production has been encouraged. Many lignocellulosic wastes have been found to successfully induce fungal laccase expression, like olive leaves (Aydinoglu and Sargin 2013) and *Eichhornia crassipes* (Wang et al. 2016b), wheat bran and kudzu vine root (Qiu et al. 2014), and apple pomace (Park et al. 2014). Agricultural waste also can supply carbon, nitrogen, and multiple mineral elements to support organism growth and laccase production (Wang et al. 2019). Moreover, the utilization of agricultural waste can reduce the use of commercial media, and thus save the production cost (Guarino et al. 2016). However, not all agricultural substrates are suitable for fungal laccase production, which requires consideration of many factors, such as nutrition sources, composition, inhibitors, and natural inducers (Wang et al. 2019). In this respect, the effect of lignocellulosic waste is well worth evaluating for the selection of appropriate support.

White-rot-fungi (WRF) are known as the main sources of laccase, but the laccase productivities from different strains vary greatly. Among various WRF, *Coriolopsis gallica* has been found with the ability to synthesize extracellular laccase efficiently in recent years, thus it is expected to become a promising laccase producer (Songulashvili et al. 2015; Xu et al. 2016). We screened a *C. gallica* strain NCULAC F1 (CCTCC M2021731) from decaying wood which generated laccase with a maximum activity of 733 U/L after 12 days of culture in potato dextrose water medium supplemented with guaiacol (Additional file 1: Fig. S1). However, this laccase activity and cultivation period are not enough ideal. To increase laccase production in a green and low-cost way, here, the possibility of common agricultural wastes as fermentation supports was assessed in comparison to the standard nutrition. Then, the generated extracellular laccase was further purified to evaluate its biochemical characteristics and catalytic performance in the removal of phenolic pollutants. Results revealed the yellow laccase produced by *C. gallica* NCULAC F1 showed great performances in the activity, stability, and catalytic ability for phenolic pollutants removal. As per best of our knowledge, yellow laccase has not been produced by *C. gallica* growing on agro-waste before this study.

## Materials and methods

### Materials

Potato dextrose water was purchased from Hope Bio-Technology Co., Ltd, Qingdao, China. 2,2′-azino-bis(3-ethylbenzothiazoline-6-sulfonic acid) diammonium salt (ABTS), phenol (PH, > 99%), 4-chlorophenol (CP, > 99%), and bisphenol A (BPA, > 99%) were acquired from Macklin Biochemical Co., Ltd, Shanghai, China. Filters of DEAE-Sepharose Fast Flow and Sephadex G-75 were purchased from Shanghai yuanye Bio-Technology Co., Ltd, Shanghai, China. All other chemical reagents used were analytical grade. Agricultural wastes (rice straw, wheat bran, sugarcane bagasse, pine sawdust, pomelo peel, navel orange peel, and rapeseed oil cake) were purchased from Taobao Mall, a Chinese online retail platform. These agro-wastes were dried and ground into powder (60 mesh).

### Microorganism and inoculum preparation

The laccase-producing strain of *C. gallica* NCULAC F1 preserved in China Center for Type Culture Collection (CCTCC M2021731) was isolated from rotten wood in Nanchang, Jiangxi Province, China, and maintained on potato dextrose agar (PDA) slant at 4 °C. Three mycelia agar plugs were inoculated into a 100 mL potato dextrose (26 g/L) medium and cultured by shaking flask at 120 rpm and 28 ± 2 °C for 5 days to prepare fungal inoculum. The final mycelia biomass was homogenized by a sterile magnetic stirrer and used as seeds for liquid fermentation.

### Optimization for laccase production

Effects of carbon source, nitrogen source, C/N ratio, and initial pH of the liquid medium on the laccase production were carried out by single-factor experiments. Initially, glucose, rice straw, sugarcane bagasse, pine sawdust, pomelo peel, and navel orange peel were separately tested as carbon sources (20 g/L), while the sole nitrogen source was ammonium sulfate. Based on the optimized carbon source, yeast extract powder, peptone, ammonium tartrate, ammonium sulfate, wheat bran, and rapeseed oil cake were further investigated as nitrogen sources (5 g/L). Subsequently, the effect of the C/N ratios (12:1,16:1, 20:1, 24:1, 28:1, and 32:1) in the optimized media (Additional file 1: Table S2) was studied by keeping the total amount of carbon and nitrogen sources to a constant (25 g/L). Afterward, the initial pH in the medium was controlled to be 3.0, 4.0, 5.0, 6.0, 7.0, and 8.0 by 1 M HCl or NaOH before sterilization to investigate its influence. The fermentation medium was composed of (g/L): carbon source (20), nitrogen source (5), KH_2_PO_4_ (3), MgSO_4_·7H_2_O (1.5), CaCl_2_ (0.5), pH 5.0. All media were sterilized for 20 min at 121 °C. Each 250 mL Erlenmeyer flask was added 100 mL medium and 3% (v/v) seeds. The cultivation was implemented on a rotary shaker at 120 rpm and 28 ± 2 °C for 7 days. The laccase activity of the culture liquid was determined every day.

### Laccase activity and protein content assays

The activity assay of extracellular laccase was determined at 25 °C by using 1.0 mM ABTS as substrate. The reaction mixture in a total volume of 3.0 mL contained 0.1 mL of laccase sample, 0.9 mL of 1.0 mM ABTS, and 2.0 mL of 100/200 mM citrate/phosphate at pH 4.5 (buffer A) solution. The increase of the OD at 420 nm in 3 min was measured by UV-9000 S spectrophotometer (Shanghai Metash Instruments Co., Ltd, China). One unit (U) of enzyme activity was defined as the amount of laccase that transformed 1 µmol ABTS per minute, and the laccase activity was expressed in the form of U/L. The soluble protein content of the enzyme sample was quantified according to Bio-Rad Protein Assay (Zheng et al. 2020) using bovine serum albumin (BSA) as a standard. A volume of 20 µL of the enzyme sample was incubated with 200 µL of Coomassie Brilliant Blue G-250 staining solution in a 96-well plate at 25 °C for 3–5 min. The absorbance was measured at 595 nm with FlexA-200 Microplate Reader (Hangzhou Allsheng Instruments Co, Ltd, China).

### Extraction and purification of the extracellular laccase

The extracellular laccase was concentrated by adding solid ammonium sulfate to 90% (w/v) saturation. Then, the precipitate from the salting-out process was dissolved and dialyzed in the 10/20 mM citrate/phosphate buffer pH 6.0 (buffer B) for desalination to obtain the crude enzyme. The crude enzyme was filtered by a 0.22 μm filter membrane and then loaded onto a DEAE-Sepharose Fast Flow column (1.0 cm × 40 cm) pre-equilibrated with buffer B. Gradient elution was figured with buffer B (pH 6.0–4.0) and NaCl (0–1.0 M) solution at 2.0 mL/min. Generally, the buffer pH needs to be 1.0 higher than the isoelectric point (pI) of the protein for effective elution. Thus, the buffer pH was controlled in the range of 6.0–4.0 as laccase pI was about 4.0 (Calvo et al. 1998). Then, all laccase-positive fractions were pooled for gel filtration chromatography performed in a Sephadex G-75 column (1.6 cm × 30 cm). The gradient elution procedure was implemented with the buffer B and NaCl (0–1.0 M) solution at a slower flow rate of 0.5 mL/min. The laccase-rich fractions were pooled, desalted, and ultra-filtrated with a 10-kDa Ultra-membrane (Millipore, USA). The purified laccase was freeze-dried and stored at 4 °C for further experiments.

### Gel electrophoresis

Sodium dodecyl sulfate polyacrylamide gel electrophoresis (SDS-PAGE) and native-PAGE were tested for the molecular mass and isozyme analysis of laccase (Calvo et al. 1998; Karp et al. 2012), respectively. The SDS-PAGE gel was executed with 12% (w/v) separating gel and 5% (w/v) stacking gel, while the native-PAGE gel was prepared with 10% (w/v) separating gel and 4% (w/v) stacking gel. After electrophoresis in the Bio-Rad Mini-Protean (Bio-Rad Laboratories, Inc, USA), the SDS-PAGE gel was colorized by Coomassie blue fast staining solution. The molecular mass of laccase was determined according to the relative migration mobility of a protein marker (11–180 kDa) that ran alongside. In native-PAGE, zymograms of laccase were visualized by submerging the gels in the buffer A solution containing ABTS, guaiacol, and 2,6-dimethylphenol, respectively for seconds to observe the number of colored bands.

### UV–vis spectrum

To investigate the absorption spectrum of laccase, the lyophilized enzyme was redissolved in deionized water and then scanned by the UV-9000 spectrophotometer in the wavelength range of 200–800 nm.

### Effect of temperature and pH on laccase activity and stability

It is well known that temperature and pH are important factors affecting the activity and stability of laccase. To evaluate the optimum temperature and pH of the purified laccase, the enzyme activity was determined at a wide temperature range (30–80 °C, 5 °C intervals) and a pH range (2.0–6.0) of buffer A, respectively. The relative activity of the laccase was calculated by considering the maximum activity as 100%. Furthermore, the laccase was incubated at high temperatures (40–70 °C) and wide pH (3.0–8.0) for 5 h to investigate its thermal stability and pH stability, respectively. The initial enzyme activity without any treatment was considered as 100% and the relative activity was calculated as a percentage.

### Effect of metallic ions, organic solvents, and inhibitors on laccase activity

To test the activity of the purified laccase toward the chemical, all treatments were executed by incubating the enzyme in the buffer A solution containing different chemicals at room temperature for 2 h. Effect of metallic ion (K^+^, Ca^2+^, Na^+^, Mg^2+^, Al^3+^, Zn^2+^, Fe^3+^, Mn^2+^, Cu^2+^, Pb^2+^, Cd^2+^, Ba^2+^) from chloride salt on laccase stability were tested at different concentrations (1.0–10.0 mM). Furthermore, the tolerance of laccase to organic solvents: methanol, ethanol, acetone, dimethyl sulfoxide (DMSO), and acetonitrile were studied at 5%, 10%, and 20% (v/v). Simultaneously, inhibition of classical inhibitors was investigated, including Tween-80 (1.0%, 5.0%, and 10.0%), dithiothreitol (DTT, 0.1, 0.5, and 1.0 mM), sodium dodecyl sulfate (SDS, 1.0, 5.0, and 10.0 mM), and ethylene diamine tetraacetic acid (EDTA, 1.0, 5.0, and 10.0 mM). After incubation with chemical reagents for 2 h, the mixed liquor was investigated the laccase activity. The relative activity was performed as a percentage of the original activity which was tested in absence of any additional chemicals (control).

### Removal of phenolic pollutants

To evaluate the catalytic performance of laccase, three common phenolic pollutants (PPs): monophenol (phenol), chlorophenol (4-chlorophenol), and bisphenol (bisphenol A) were selected as the target PPs. Freeze-dried laccase was dissolved in water to prepare a solution with the laccase activity of 80 U/mL. Considering the optimum temperature, pH, and stability of CGLac, the degradation experiments were tested at 50 °C and pH 4.5. Laccase solution (1:10, v/v) was added into the buffer A solution containing one of PPs at 50 mg/L, oscillating at 50 °C and 150 rpm in a shaking bath for 120 min. The control was treated in the absence of laccase. The PPs concentration was determined by the colorimetric method (*λ*_max_ = 500 nm) described by Sadeghzadeh et al. (2020) with minor modification. The removal efficiency was calculated by Eq. (1). Subsequently, the degradation processes of three phenolic pollutants were analyzed by the first-order kinetic model (Eq. 2) and second-order kinetic model (Eq. 3). The equations of removal efficiency and kinetic models were given below:

1 $$Removal\;efficiency \;(\%) =({C}_{0}-C)/{C}_{0} \times 100\%$$

2 $$ln(C/{C}_{0})=-kt$$

3 $$1/C=1/{C}_{0}+kt$$

where *C* was the residual concentration of PPs (mg/L) at time *t*, *C*_0_ was the initial concentration of PPs (mg/L), *t* was reaction time (min), and *k* was kinetic constant (h^−1^).

### Statistical analysis

SPSS (IBM, 26.0) was used for statistical analysis with a one-way analysis of variance (ANOVA). The experimental data were obtained from three replicate tests and expressed as mean ± SD (n = 3). Data plots were performed by Origin 2018.

## Results

### Enhancement of laccase production by *C. gallica* in low-cost medium

**Fig. 1:**
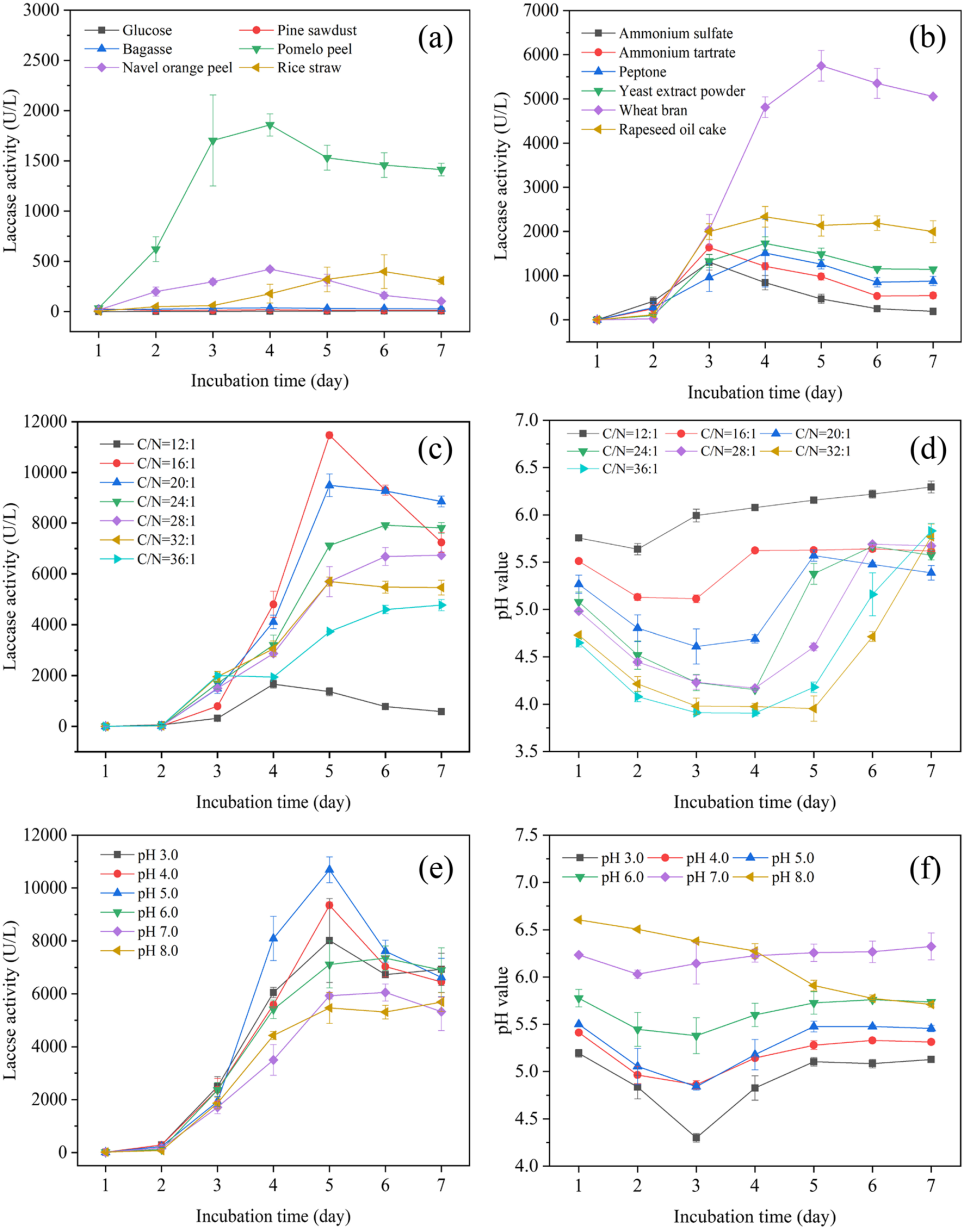
Laccase production and pH variation in culture liquid under different culture conditions. Effect of carbon source (**a**), nitrogen source (**b**) on laccase production. Effect of C/N ratio on laccase production (**c**) and pH variation (**d**). Effect of the initial pH on laccase production (**e**) and pH variation (**f**)

Although a lot of literature has identified the positive role of agricultural substrates in laccase production, the effects of agro-wastes are different from the types of waste used. When agro-wastes were used as carbon sources, as shown in Fig. 1a, pomelo peel showed the most positive influence on laccase expression (1857 U/L), and then followed by navel orange peel (422 U/L), rice straw (398 U/L), bagasse (36 U/L), and pine sawdust (17 U/L). The lowest laccase activity (only 4 U/L) appeared in the culture with glucose. Glucose is the standard carbon source, but it showed poorer performance than agricultural residues in fungal laccase production in our research. The highest laccase activity (1857 U/L) from *C. gallica* obtained in the fermentation with pomelo peel was higher than that of another laccase from *Trametes pubescens* grown on the ground mandarin peelings (228 U/L) (Osma et al. 2007a) and banana skin (Osma et al. 2007b). Pomelo peel had been confirmed to be rich in polysaccharides, flavonoids, phenolic acids, and various nutrient substances (Tocmo et al. 2020). Thereby, the good performance of pomelo peel in laccase production may relate to these multiple nutrients and inducers, which can support fungal growth and stimulate laccase synthesis simultaneously. Sharma et al. (2017) also suggested that the laccase inducers in citrus fruit peel might be various phenolics and flavonoids. In addition, the lignin and cellulose in the peels of the fruits probably stimulate laccase production (Rosales et al. 2005). Therefore, fruit waste (especially pomelo peel) is a promising substrate for fungal laccase production. Taken together, the pomelo peel was a great source of carbon and inducer for laccase production.

As regards the effect of nitrogen sources (Fig. 1b), similarly, the nitrogen-rich by-products, wheat bran (5750 U/L) and rapeseed oil cake (2332 U/L) also exerted a greater influence on laccase synthesis than the other four commercial nitrogen sources (1300–1800 U/L). This strong influence of agricultural substrates might be due in part to the stimulatory effect of containing natural inducers. Additionally, in comparison with the initial cultivation (Additional file 1: Fig. S1), the culture period of *C. gallica* was shortened from 12 days to 5 days when using both pomelo peel and wheat bran. It showed that the mixed substrates could greatly shorten the production time. Therefore, the co-fermentation pattern with the pomelo peel and wheat bran shows attractive potential in laccase production in a green and low-cost way.

In addition to nutrient sources, the C/N ratio and initial pH value also affected fungal growth and thus laccase production. According to the elemental composition of C and N in the optimal substrates (Additional file 1: Table S1), the effect of various C/N ratios (12:1 to 36:1) was tested by varying the amount of pomelo peel (C-source) and wheat bran (N-source) under the condition of initial pH 5.0. As shown in Fig. 1c, when wheat bran was used as the sole substrate (C/N ratio about 12:1), the activity peak (1522 U/L) was far lower than that of other mixed substrates with the higher C/N ratios (16:1–36:1). A possible reason was that natural inducers from the mixed substrates presented a synergistic effect but not the sole wheat bran. In addition, low laccase activity at C/N ratio of 12:1 might also be associated with the poor growth under overhigh pH (> 5.5), because other higher C/N ratios caused pH to be lower than 5.5 (Fig. 1d). With the increase of C/N ratio from 16:1 to 36:1, the peak value of laccase activity decreased from 11,468 U/L to 4773 U/L (Fig. 1c). However, the enzyme activity after 5 days of cultivation at C/N ratio of 16:1 decreased from the highest activity of 11,468 U/L to 7243 U/L. The main reason for the decrease of enzyme activity after 5 days of cultivation at C/N ratio of 16:1 (Fig. 1c) was the organism entering into decay. In this period, nutrients were depleted, metabolic waste accumulated, and enzyme synthesis was affected. Similarly, laccase activity also showed a decreasing trend (from 9490 U/L to 8855 U/L) after 5 days of cultivation at a C/N ratio of 20:1 (Fig. 1c). Taken together, C/N ratio of 16:1 was a suitable condition for fungal growth and laccase production.

Based on the above optimization results, the initial pH of the medium was examined further in a wide range from 3.0 to 8.0. All treatments were observed with a similar profile: laccase activity appeared on day 2, attained the peak value on day 5, then decreased at the end of culture (Fig. 1e). The laccase production profiles revealed that initial pH did not impact the peak time of laccase production, but the value of enzyme activity. Therein, the highest and lowest laccase activity reached 10,690 U/L and 5465 U/L when the initial pH was 5.0 and 8.0, respectively. The pH environment can affect the growth of the microorganism, while the metabolic activity of microorganisms also can affect pH variation. And the change in pH value can reflect the activity of the fungi. Therefore, the changes in pH values were also studied during the process of liquid fermentation. According to the profiles of pH variation during liquid fermentation (Fig. 1d, f), we found that pH values during cultivation kept in the range of 5.0 to 5.5 under the conditions with a C/N ratio of 16: 1 and initial pH of 5.0. Under these conditions, the laccase activity was highest. This result suggested that the optimal initial pH for CGLac production was about 5.0, and the pH should be kept constant (5.0–5.5) during cultivation. Previous studies also proposed that the weakly acidic environment was beneficial to fungal laccase production (Ire and Ahuekwe 2016; Mathur et al. 2013).

### Purification of the extracellular laccase from *C. gallica*

**Fig. 2:**
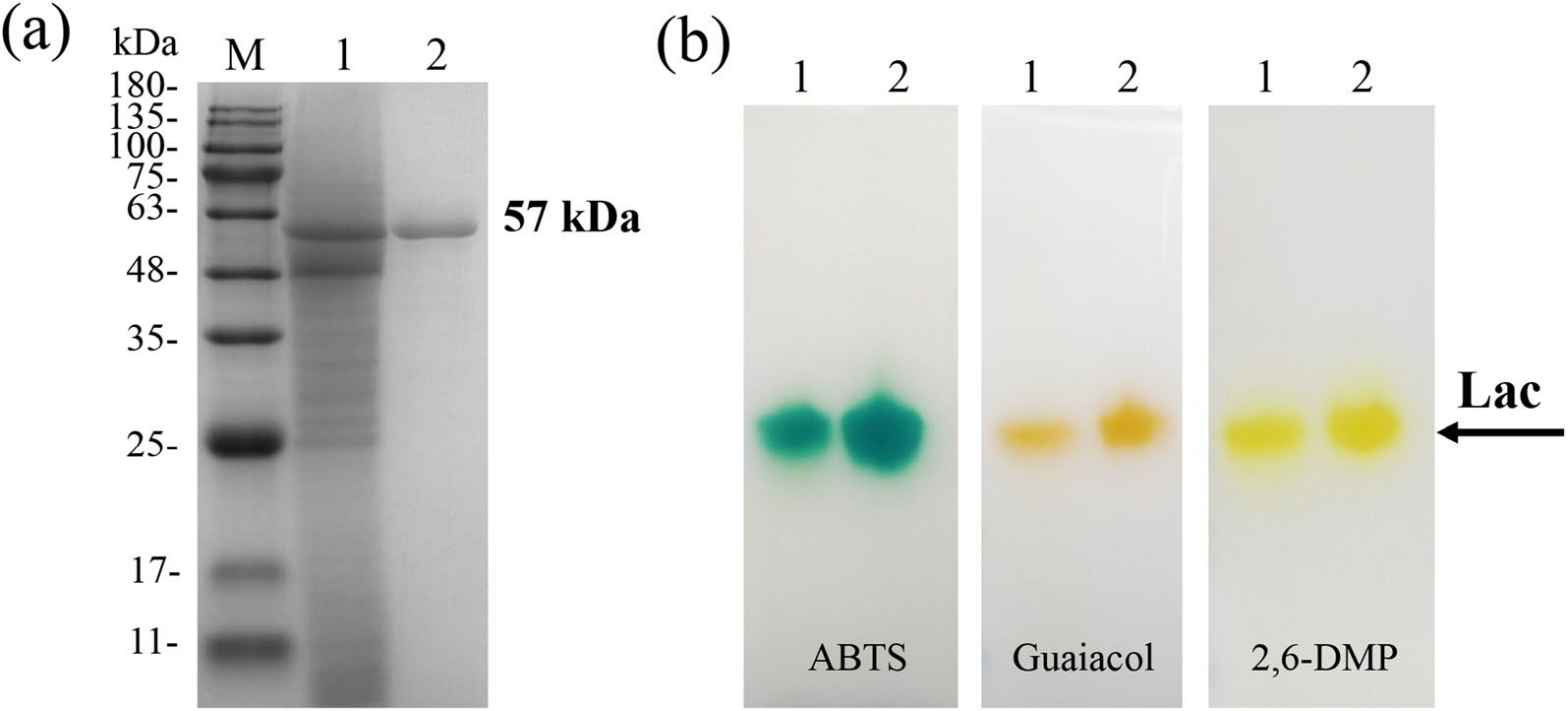
SDS-PAGE (**a**) and native-PAGE (**b**) electrophoretograms of the laccase from *C. gallica *(lane M: molecular mass of marker; lane 1: the crude laccase; lane 2: the purified laccase; kDa, the unit of protein molecular mass; ABTS, guaiacol, and 2,6-DMP were used as substrates)

Extracellular laccase produced in the optimized medium was separated and purified through salting out, anion exchange chromatography, and gel filtration. Results showed that four protein peaks and a laccase peak were separated from the eluent of the anion exchange column (Additional file 1: Fig. S2), while a symmetric protein peak conformed to a laccase peak was obtained after further purification by gel filtration (Additional file 1: Fig. S3). One protein band was observed on the SDS-PAGE gel in the laccase sample purified by gel filtration (Fig. 2a). These results implied that the crude laccase had been purified effectively. After three steps of purification, the specific activity of laccase increased to 188.79 U/mg protein, purification fold of 6.13 and recovery of 45.64% (Table 1). These data are better than the purification results of another *C. gallica* laccase, which obtained a specific activity of 154.7 U/mg protein, 5.5-fold purification and a yield of 16.2% (Songulashvili et al. 2016). Although multi-step purification has been developed to promote purity, it often leads to the severe loss of enzyme activity. A quintessential example should be cited was that laccase from *Trichoderma harzianum* was purified 25 folds, but only 7% recovery was obtained after five procedures (Bagewadi et al. 2017). Therefore, the loss of enzyme and its activity should not be neglected in purification.

**Table 1:** Results of the purification of extracellular laccase from *C. gallica*

Purification procedure	Total activity (U)	Total protein (mg)	Specific activity (U/mg)	Purification fold	Percent recovery (%)
Culture supernatant	20150.00 ± 624.61	654.50 ± 13.06	30.78 ± 0.34	100.00 ± 3.10	1.00 ± 0.01
Ammonium sulfate precipitation (90%)	18806.67 ± 358.27	537.49 ± 2.18	34.99 ± 0.81	93.33 ± 1.78	1.14 ± 0.03
Anion exchange (DEAE-FF)	9691.67 ± 162.63	69.78 ± 0.14	138.88 ± 2.06	48.10 ± 0.81	4.51 ± 0.07
Gel-filtration (Sephadex G-75)	9195.67 ± 89.10	48.71 ± 0.27	188.79 ± 2.88	45.64 ± 0.44	6.13 ± 0.09

### Molecular mass and isoforms of the laccase from *C. gallica*

Most fungal laccases have molecular weights in the range of 50–70 kDa (Rivera-Hoyos et al. 2013). According to the result of SDS-PAGE, *C. gallica* laccase (CGLac) showed a molecular mass of approximately 57 kDa (Fig. 2a, lane 2). The molecular mass of CGLac was in accordance with that of other *C. gallica* laccases shown in Table 2. In native-PAGE, the enzyme catalyzed substrates to colored oxidation products, and the number of colored bands represented the number of laccase isoforms (Othman et al. 2018). According to the zymography of laccase, only one colored band appeared both in the crude and purified laccase samples, which indicated that only one laccase was detected (Fig. 2b). Although laccase isoforms were previously found in *C. gallica* (Calvo et al. 1998), this discrepancy illustrated the presence and number of laccase isoforms probably depended on culture condition and strain used. Of special note was that the active enzyme not only catalyzed ABTS into green oxidate (Fig. 2b, lane 1), but also catalyzed guaiacol and 2,6-dimethylphenol into brown oxidate (Fig. 2b, lane 2) and yellow oxidate (Fig. 2b, lane 3), respectively.

**Table 2:** Characteristics of laccases from different *C. gallica* strains

*C. gallica* strain	Molecular mass (kDa)	Optimal temperature (°C)	Optimal pH^a^	Thermal stability range (°C)	pH stability range	References
NCULAC F1	57	60	2.5	40–60	6.0–8.0	This study
1184	66	70	2.5–3.0	NA	NA	Songulashvili et al. (2016)
T906	53.7	60	2.5	20–60	2.0–4.0	Xu et al. (2016)
UAMH 8260	66	65	3.8	40–60	5.5–9.0	Vandertol-Vanier et al. (2002)
A-241	84.1	70	3	28	4.5	Calvo et al. (1998)

*NA* not available

^a^The pH optima of laccase were determined with ABTS as substrate

### Spectral property of CGLac

**Fig. 3:**
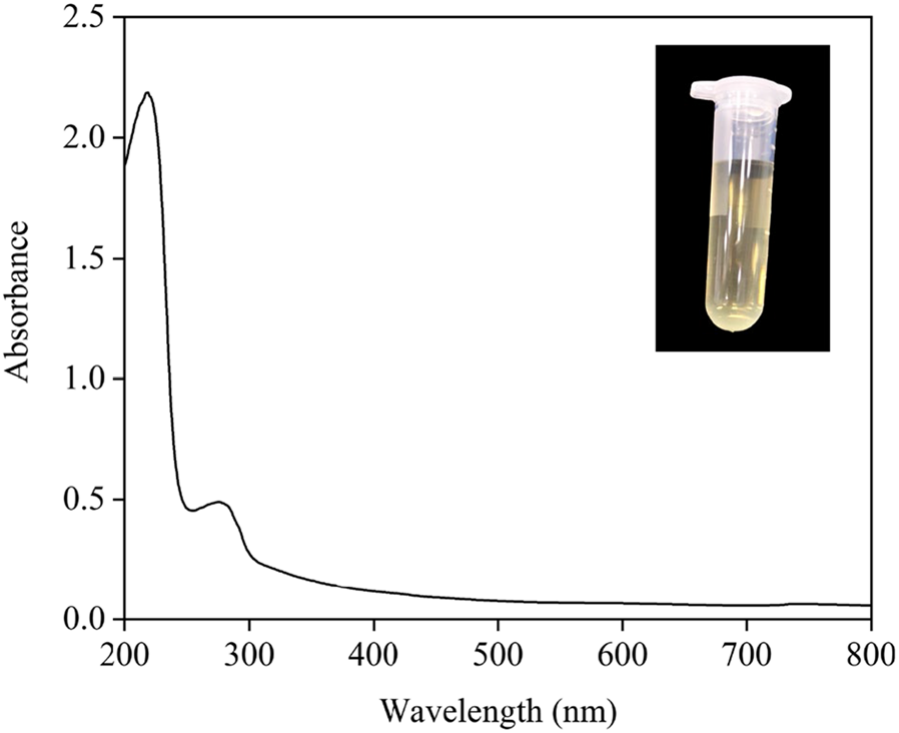
The UV–vis absorption spectrum of purified CGLac, in 10/20 mM citrate/phosphate buffer pH 6.0

In general, a typical laccase molecule contains four copper atoms with three types of copper sites (T1, T2, and T3), both type I and type III copper ions have the characteristic absorption in the UV–vis spectrum except for type II (Dwivedi et al. 2011; Rivera-Hoyos et al. 2013). Herein, the absorption peak at 610 nm represents the T1 copper site of typical blue laccase, while the shoulder around 330 nm represents the T3 copper site of laccase (Radveikienė et al. 2021). The UV–vis spectrum of CGLac performed a characteristic absorption peak of protein near 280 nm, but no peak near 610 nm and 330 nm (Fig. 3). This result revealed CGLac had unusual spectral characteristics compared with blue laccase. In addition, the purified CGLac solution had a yellow color (shown in Fig. 3). These results suggested that CGLac might be a yellow laccase rather than the typical blue one.

### The optimal temperature and thermostability of CGLac

**Fig. 4:**
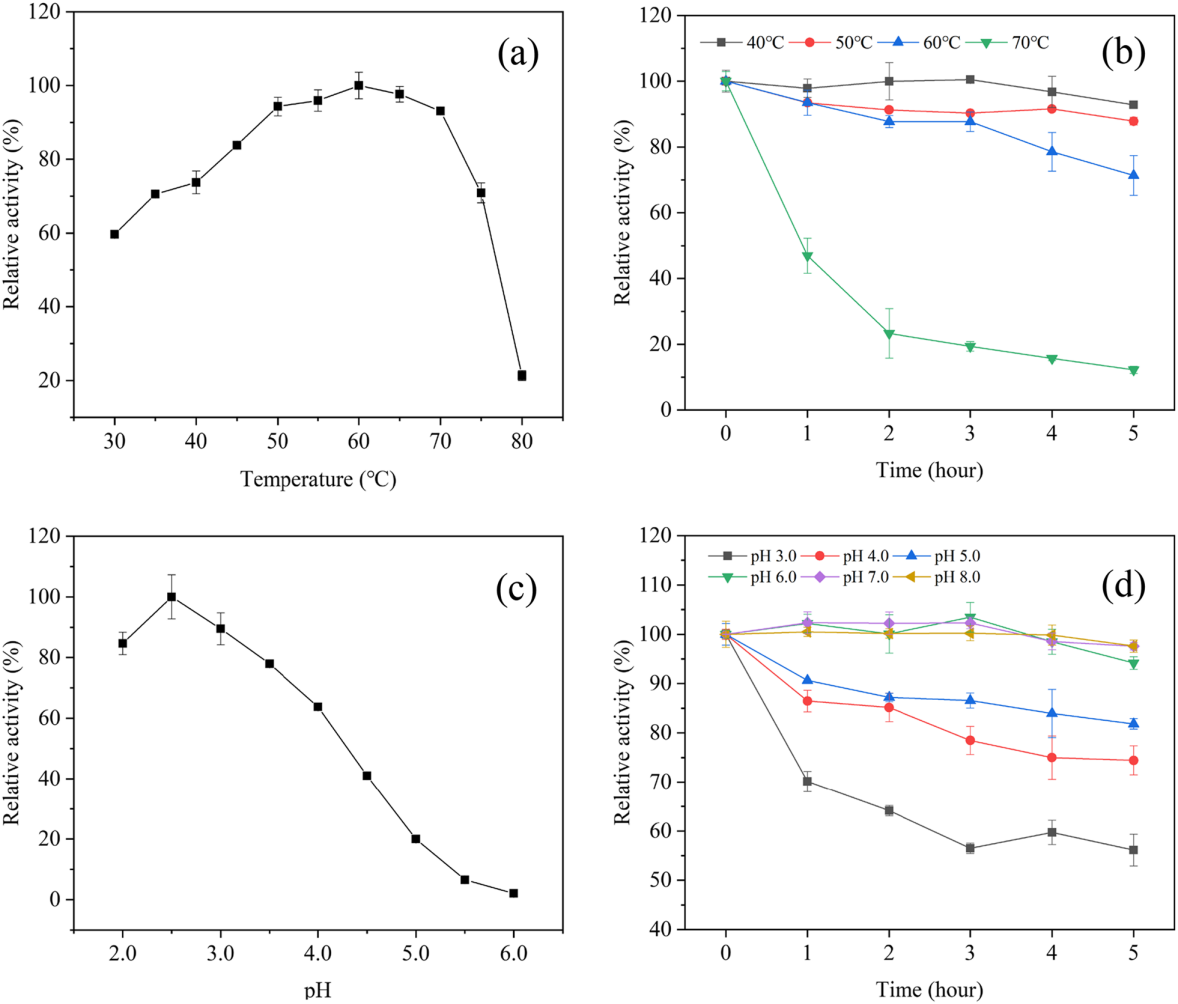
Effect of temperature and pH on the activity and stability of CGLac by using 1.0 mM ABTS as a substrate. **a** The optimal temperature of CGLac. **b** Thermostability of CGLac at 40–70 °C. **c** The optimal pH of CGLac. **d** The pH stability of CGLac at pH 3.0–8.0.

The effect of temperature on the activity of CGLac was shown in Fig. 4a. CGLac maintained highly relative activity (> 60%) at a wide range of 30–75 °C but reduced drastically above 80 °C. The thermal inactivation links to the structural stability of the laccase because high temperatures can directly change the secondary structure of laccase and thus alter laccase stability (Li et al. 2021). Nevertheless, CGLac showed high activity within the range from room temperature to high temperature. The optimal temperature of CGLac was 60 °C, which was consistent with other *C. gallica* laccases shown in Table 2. Thermal stability is one of the important properties of an enzyme for catalysis. As shown in Fig. 4b, when CGLac was incubated at 40 °C, 50 °C and 60 °C for 5 h, the relative activity was 92.80%, 87.85%, 71.36%, and the half-live (*t*_1/2_) was nearly 60 h, 34 h and 11 h, respectively. CGLac was even retained around 50% of initial activity at 70 °C in the initial 1 h. This good thermostability is desirable and content for CGLac to exert catalytic performance in industrial applications.

### The optimal pH and pH-stability of CGLac

Generally, fungal laccases are acidic proteins and fairly active in the pH range of 2.0–5.0 (Baldrian 2006). As shown in Fig. 4c, the laccase was highly active in a narrow pH range between 2.0 and 4.0, presenting a similar optimal pH (2.5) to that of other *C. gallica* laccases (Table 2). The activity of CGLac was easily inactivated at a pH over 6.0, which may be the result of OH^−^ inhibition on the T2/T3 copper sites, disrupting the internal electron transport from the T1 to the T2/T3 centers in the laccase molecule (Xu 1997). CGLac was unstable at lower pH conditions, finally decreasing to 81.83%, 74.44%, and 56.15% when treating the enzyme at pH 5.0, 4.0 and 3.0 for 5 h, respectively (Fig. 4d). Differently, CGLac maintained over 90% relative activity after treatment of pH 6.0–8.0 for 5 h. These data suggested that the laccase was more stable under neutral and alkaline pH regions (pH 6.0–8.0) than acid conditions (pH < 6.0).

### Tolerance of CGLac against metal ions

**Fig. 5:**
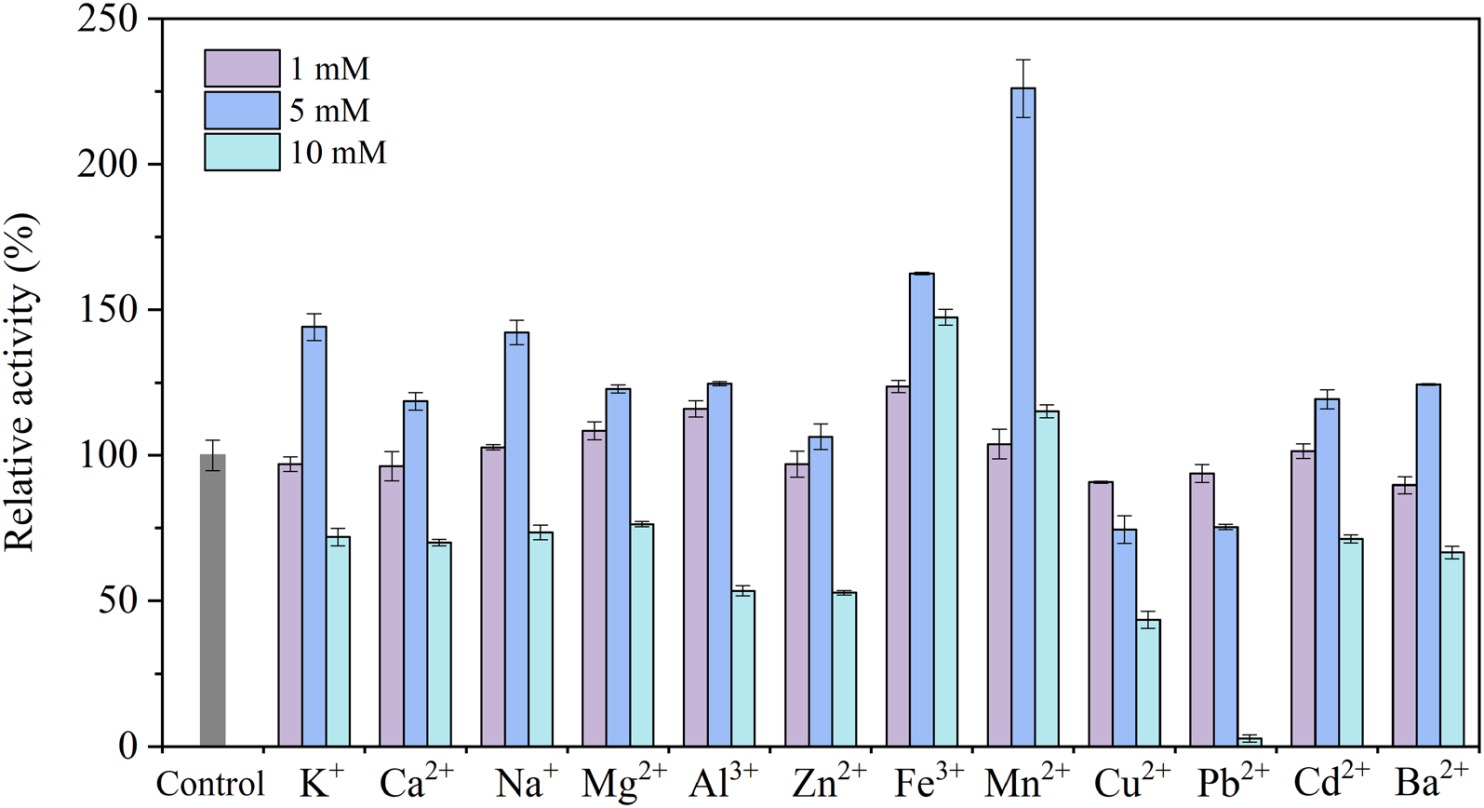
Effect of various metal ions on CGLac activity

The effect of metal ions on CGLac stability was tested at different concentrations (1.0–10.0 mM) for 2 h (Fig. 5). The effect of metal ions on CGLac was found to be highly concentration-related. The main trend was that metal ions had no obvious effect at 1.0 mM, had a stimulatory effect at 5.0 mM, and had an inhibitory effect at 10.0 mM. Exceptionally, the activity of CGLac was inhibited by Ba^2+^ (10.26%) and stimulated by Fe^3+^ (23.69%) at 1.0 mM; slightly inhibited by Cu^2+^ (25.49%) and Pb^2+^ (24.65%) at 5.0 mM; stimulated by Fe^3+^ (47.40%) and Mn^2+^ (15.21%) at 10.0 mM. Notably, the activity of CGLac was highly increased to 162.56% and 226.05% in the presence of Fe^3+^ and Mn^2+^ at 5.0 mM, respectively. Similar activation by Mn^2+^ (5–10 mM) also was reported in laccase from *Sporothrix carnis* (Olajuyigbe and Fatokun 2017) and *Thielavia* sp. (Mtibaa et al. 2018). However, potent inhibition by Fe^3+^ and Mn^2+^ was found in *Bacillus licheniformis* laccase at 5 mM (Li et al. 2021). Likewise, Wang et al. (2017) also reported that 10 mM Fe^3+^ seriously inhibited over 90% activity of the laccase from the fungus *Cerrena unicolor* GSM-01. This effect probably was caused by the interaction between metal ions and the active site of the enzyme, thereby changing the stability of the laccase (Mehandia et al. 2020). In summary, the inhibitory or stimulatory effect of metal ions on laccase activity might be implicated to not only the type and concentration of metals used but also the source of laccase.

### Tolerance of CGLac against organic solvents and inhibitors

**Fig. 6:**
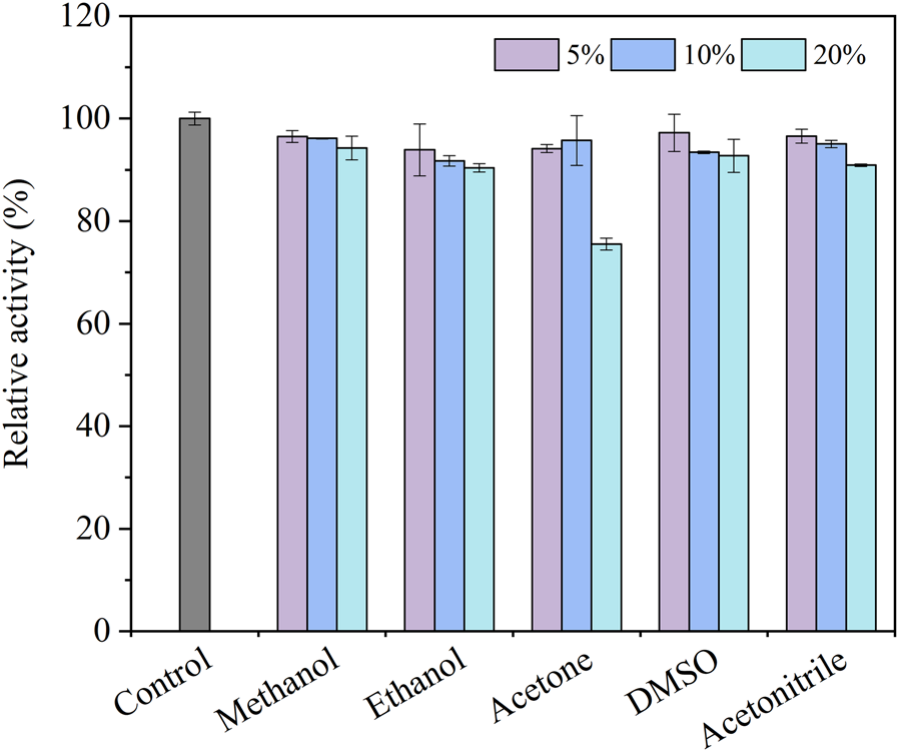
Effect of common organic solvents on CGLac activity

Since organic solvents are important media in the non-aqueous system and industrial hazards in the environment, investigation of the tolerance of CGLac to organic solvents will be helpful for the biotechnological application of the enzyme. In this study, five common organic solvents were tested at a concentration from 5 to 20% (v/v). After incubation with each solvent for two hours, CGLac was slightly inhibited with the increase of organic solvent concentration (Fig. 6). Laccase retained its activity above 93% in the presence of all tested organic solvents at the concentration of 5%. A slight decrease in CGLac activity was found when exposed to up to 20% (v/v) of organic solvent except for acetone, which caused 24.45% inhibition.

**Fig. 7:**
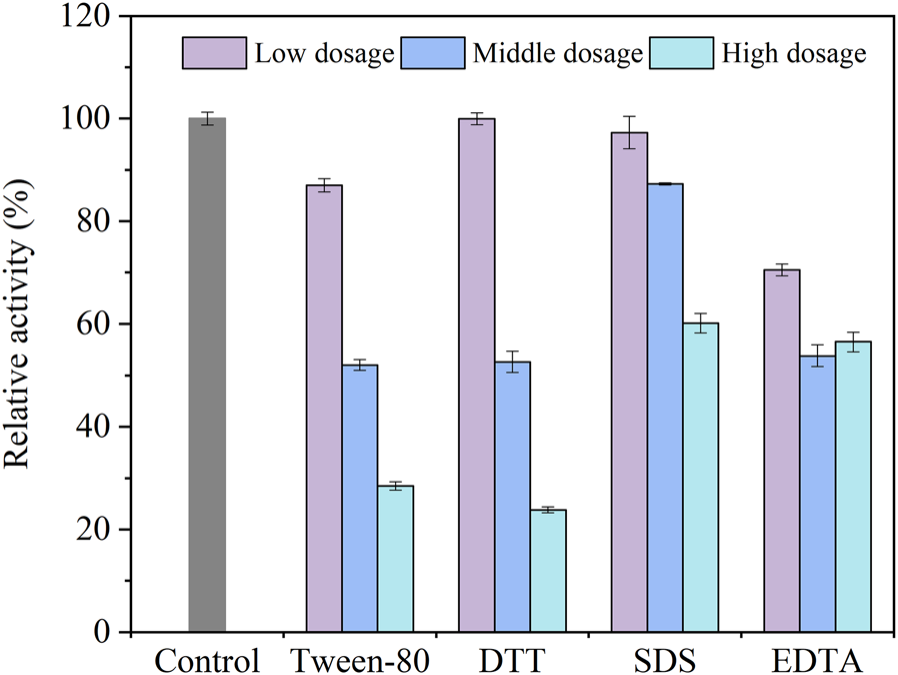
Effect of putative inhibitors on CGLac activity (Tween-80 concentrations were 1.0%, 5.0% and 10.0%, respectively; DTT concentrations were 0.1, 0.5, 1.0 mM, respectively; SDS concentrations were 1.0, 5.0, 10.0 mM, respectively; EDTA concentrations were 1.0, 5.0, 10.0 mM , respectively)

Exposure of laccase to harsh conditions, such as some putative inhibitors, commonly causes the loss of activity. Tween-80 (detergent), DTT (reductant), SDS (surfactant), and EDTA (sequestrant) were investigated at a series of concentrations, and the inhibitory effect on the CGLac activity was enhanced with the increase of tested inhibitor concentration (Fig. 7). Low concentration of Tween-80 (1.0%), DTT (0.1 mM), and SDS (1.0 mM) had slight inhibitory effects on the CGLac activity. In the presence of 10.0% Tween-80 and 1.0 mM DTT, the relative activity of CGLac decreased by 71.51% and 76.16%, respectively. About the chelator agent EDTA and surfactant SDS, they caused a relatively milder inhibition, resulting in only about 40% loss of laccase activity even at 10.0 mM. This slight inhibitory effect is similar to that obtained for the laccase from *Sporothrix carnis* (Olajuyigbe and Fatokun 2017), which retained around 60% of its activity at 10 mM of SDS and EDTA.

### Application of CGLac for phenolic pollutants removal

**Fig. 8:**
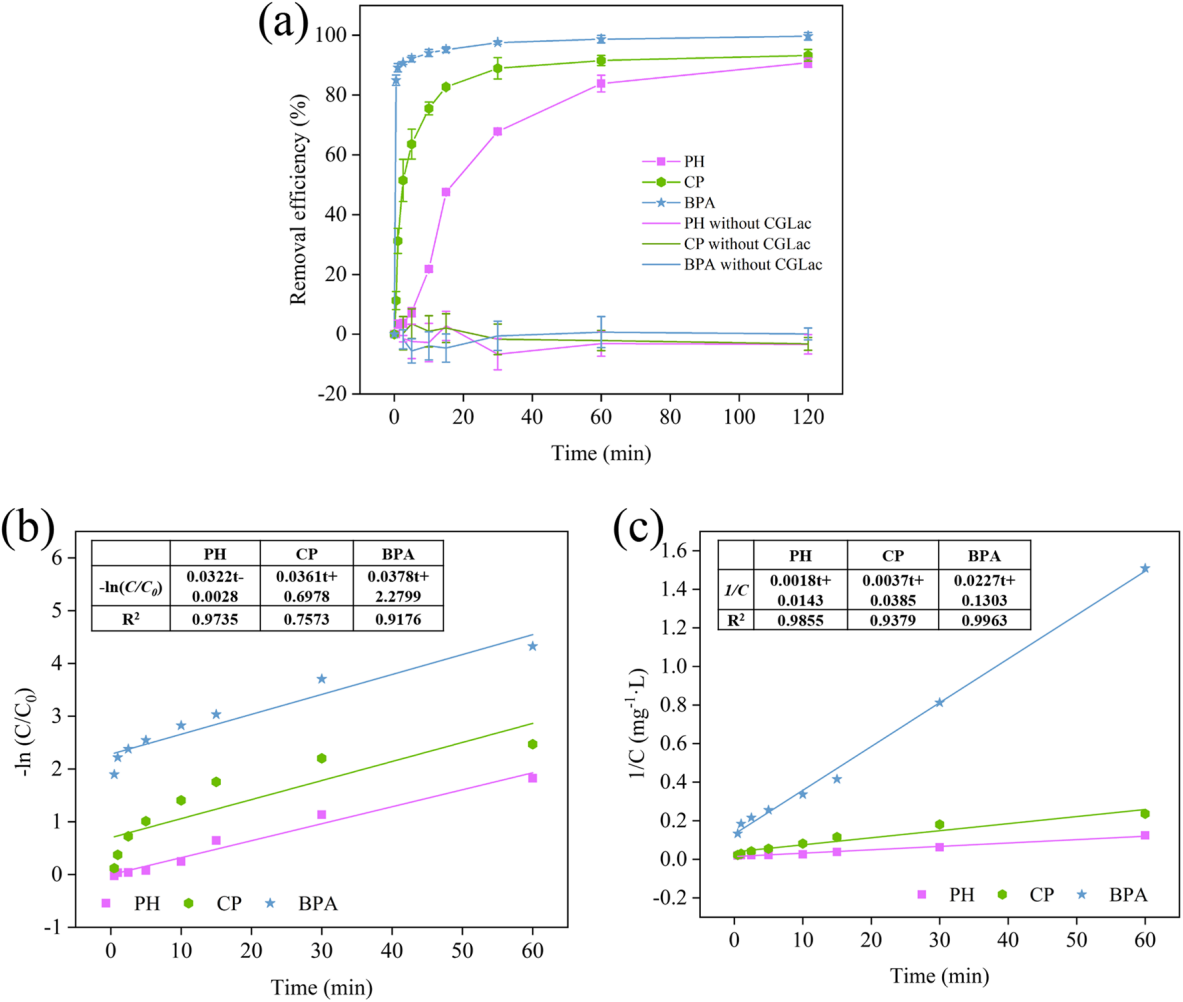
**a** Removal efficiency of phenol (PH), 4-chlorophenol (CP), and bisphenol A (BPA) by CGLac. The first-order kinetic model (**b**) and second-order kinetic model (**c**) of PPs removal processes

As a “green tool” in biotechnology, laccase can degrade a variety of toxic phenolic pollutants (PPs) present in industrial effluents. In this study, phenol (PH), *p*-chlorophenol (CP), and bisphenol A (BPA) was selected as the target PPs to access the application potential of CGLac. With CGLac added, PPs were removed rapidly in the first 60 min, and the removal rates were higher than 80% (Fig. 8a). When the reaction time increased to 120 min, the final removal rates of PH, CP, and BPA reached 90.78%, 93.26%, and 99.66%, respectively. The content of PPs remained constant in the control without enzyme addition, thus the laccase was the only one responsible for PPs removal. For understanding the removal profiles of PPs, the laccase-mediated kinetic processes in the period of the first 60 min were demonstrated by the first-order and second-order kinetic models (Fig. 8b, c). Comparing the results of the two models, it was found that the *R*^2^ values obtained in the second-order kinetic model (*R*^2^ = 0.93–0.99) were greater than those of the first-order kinetic model (*R*^2^ = 0.75–0.97), which indicated that the laccase-mediated removal of PPs might follow the former.

## Discussion

In this study, we improved the laccase production of *C. gallica* by using low price substrates as natural substrates and inducers. We found that fruit peelings and rice straw showed a more positive effect on laccase production than bagasse and pine sawdust. This discrepancy may be due to their different trophic component. Generally, glucose had been perceived as the standard carbon source for microbes, but it led to lower laccase production than agricultural residues in our research. Similarly, the nitrogen-rich by-products, wheat bran, and rapeseed oil cake also exerted a greater effect on laccase synthesis than commercial nitrogen sources. These results revealed that agricultural substrates showed better performances in increasing laccase expression than commercial carbon and nitrogen sources, which might be due to the stimulatory effect of natural inducers. Thus, this finding identifies the importance of induction for fungal laccase generation.

Previous studies routinely added standard nutrition (e.g., glucose and yeast extract) into the substrate media to support the initial fungi growth (Osma et al. 2011; Songulashvili et al. 2015). Differently, in this work, the agricultural substrates were used to completely replace the standard carbon and nitrogen sources, and the achieved cultivation period (5 d) was much shorter than that of previous studies (10–13 d). Moreover, after the successive optimization, the highest laccase activity was 10,690 U/L obtained on day 5, which was finally increased by 13.58 folds of activity and shortened by 7 days of culture from the initial culture (733 U/L, 12 days, Additional file 1: Fig. S1). These data were better than that of another *C. gallica* strain, which obtained a 3.2-fold increase in laccase activity (4880 U/L) after 10-day cultivation with sawdust waste (Daassi et al. 2016), as well as better than a well-studied fungus, *Trametes versicolor*, which was reported with lower laccase activity (5005.55 U/L) and longer period (12 days) in the fermentation of cotton gin wastes and vinasse (Pinheiro et al. 2020). Agrawal and Verma (2020) reported that the activity of yellow laccase from *Stropharia* sp. increased to 323.2 U/L after 16-day cultivation in the media with glucose and yeast extract. Compared with the research of Agrawal and Verma (2020), our results obtained higher laccase activity in a shorter time and cheaper production. Therefore, it’s a feasible, efficient, green, and economical avenue for the enhancement of fungal laccase production by using the low-cost agricultural residues of pomelo peel and wheat bran.

Blue laccases are reported to be more frequent than yellow laccases in most fungal laccases. To be present, only several fungi have been found to generate yellow laccases, like *Aureobasidium pullulans*, *Leucoagaricus gongylophorus*, *Lentinus squarrosulus*, *Stropharia* sp., *Trametes* sp. (Ademakinwa and Agboola 2016; Agrawal and Verma 2020; Ike et al. 2019; Mukhopadhyay and Banerjee 2015; Wang et al. 2018). In the present study, CGLac was yellow and had no typical blue laccase spectra. To our best knowledge, yellow laccase from *Coriolopsis gallica* has not been reported to date. Leontievsky et al. (1997) proposed that the formation of yellow laccases was the result of the binding of lignin-derived molecules to enzyme protein. In addition, the types of laccases probably relate to strains and growth conditions. Radveikienė et al. (2021) reported *Botrytis cinerea* 241 secreted two different laccases (blue and yellow laccases) depending on the cultivation conditions. They found the medium with lower copper concentration was more efficient for yellow laccase production.

There is now a consensus that temperature and pH are the important factors affecting the activity and stability of laccase. CGLac presented optimal activity at 60 °C and pH 2.5 and showed good stability in the range of 40–60 °C and pH 6.0–8.0. These properties are similar to that of the laccases from other *C. gallica* strains (Table 2). One of the main limits to the rapid development of laccase in industrial applications is the requirement for enzymatic stability under high temperatures. CGLac showed better thermostability with a 6.9-time *t*_1/2_ at 60 °C (11 h) and 12-time *t*_1/2_ at 70 °C (1 h) than another thermostable laccase from *Trametes trogii* that reported with a *t*_1/2_ of 1.6 h at 60 °C and 5 min at 70 °C (Ai et al. 2015). This good thermal stability is beneficial for CGLac to exert well catalytic performance in thermal processes. In terms of pH stability, CGLac was stable in the neutral region from pH 6.0 to 8.0. A similar finding was discovered in the research of Ramírez-Cavazos et al. (2014), which found the laccase from *Pycnoporus sanguineus* was highly active at pH 2.0–4.0 and was stable at pH 6.0–8.0. This result revealed that laccase purification, storage, and application should be done in a neutral pH environment as far as possible. However, this pH stability would limit enzyme application in the challenging acidic environment, so the acid resistance of CGLac needs to be improved by some proposed strategies in further work.

Tolerance to the chemical reagents (metal ions, organic solvents, and protein inhibitors) also is a necessary property for laccase application in biotechnological fields, since various chemicals may be present in the environment that adversely affect laccase. Commonly, inhibition of laccase activity by metal ions was enhanced with the increase of ion loads (Ezike et al. 2020). The effect of metal ions on the activity of CGLac was also found to be metal type-related in this study. Notably, CGLac was highly activated in the presence of Mn^2+^, whereas, severely inhibited by Pb^2+^. Since fungal laccases are multicopper oxidases, laccase activity can be affected by Cu^2+^ with various (stimulatory, inhibitory, or neutral) effects (Wang et al. 2017). CGLac was inhibited by copper ions at the tested concentrations of 1.0–10.0 mM, and the inhibition intensified with the increase of copper concentration. Ai et al. (2015) reported slight activation of the laccase from *T. trogii* YDHSD by cupric ions at a low concentration of 1 mM. Differently, Olajuyigbe and Fatokun (2017) observed that the laccase from *Sporothrix carnis* CPF-05 was inhibited by cupric ion at low concentrations of 1 mM and 5 mM, but was stimulated by Cu^2+^ at a higher concentration of 10 mM, they stated copper ions filled T2 copper binding sites of laccase and thus resulted in the activation of laccase.

The loss of laccase activity easily was caused by polar organic solvents and inhibitors (Olajuyigbe and Fatokun 2017). There are two reasonable explanations for the inhibition of organic solvents: mixed solutions result in a variation of the effective pH (Rodakiewicz-Nowak et al. 2000), moreover, polar solvents deprive the “essential water” from the laccase surface, and also change the conformational of laccase (Wang et al. 2016a; Zhang et al. 2020b). Differently, CGLac maintained highly relative activity in the presence of methanol, ethanol, acetone, DMSO, and acetonitrile at the concentration range of 5–20% (v/v). Another yellow laccase from *Trametes polyzona* also displayed high relative activity in the presence of these organic solvents (Ezike et al. 2020).

Enzymes are also sensitive to chemical inhibitors generally. As the dosage of the trial inhibitor increased to the highest, laccase activity was inhibited gradually, among which the most magnitude of inhibition was obtained in DTT. This strong inhibition of reductant DTT was attributed to its reductive effect on the disulfide bridges (impacting the stability of laccase structure) and the reduction of the oxidized substrate by the sulfhydryl groups of DTT (Safary et al. 2016). The slight inhibition of EDTA may be due to its low accessibility to the active copper sites (essential to laccase catalysis), while the effect of SDS could be explained by the partial unfolding of compact protein structure, improving the hydrophobic interactions between the substrate and the enzyme active site (Mtibaa et al. 2018). Compared with many bacterial laccases (Li et al. 2021; Mehandia et al. 2020; Safary et al. 2016), CGLac showed better resistance to the putative inhibitors. The stability of laccases possibly relates to their native sensitivity to inhibitors. In a word, CGLac shows great biochemical characteristics (high tolerance toward thermal, salt, solvents, and inhibitors), appearing its applied potential in the complicated environment, like chemical industry wastewater.

The degradation of PPs is one of the attractive applications of laccase in biotechnological fields. In the present study, we found CGLac had the highest removal efficiency of BPA and then followed by CP and PH. BPA was removed fast in the initial minutes, which was comparable with the result of Fu et al. (2019) in which the degradation of BPA was completed for 5 min, while a longer removal time was reported by Zhang et al. (2020a) about 4 h (100% removal) and Ran et al. (2019) about 12 h (85% removal). According to previous studies, the removal effect of laccase on PPs is also different. For example, 78% and 84% of phenol and *p*-chlorophenol were removed by the laccase from *Trametes versicolor*, respectively (Liu et al. 2012), and the removal percentage of phenol and bisphenol A also by *T. versicolor* laccase were 96.4% and 85.5%, respectively (Tarasi et al. 2018). It has been proved that phenolic compounds could be oxidated by laccase into insoluble oligomeric or polymeric precipitate which can then be removed by physical filtration (Kudanga et al. 2011; Ran et al. 2019). This implies that PPs could be removed easily from the aqueous solution thus achieving water purification. In addition, a new study reported this polymerization of PPs by laccase can produce valuable humic constituents (Li et al. 2022). Therefore, laccase has huge potential in the treatment of PPs.

In conclusion, we found a high-performance yellow laccase from *C. gallica*, and explored its production strategy and enzymatic characteristics. The findings highlighted the co-fermentation of pomelo peel and wheat bran effectively promoted the production of CGLac and shortened the fermentation period synchronously. The utilization of agricultural wastes in fermentation substrates could reduce the stress of waste management, as well as the enzyme production cost. Additionally, CGLac showed good properties of high activity, and stability and showed high catalytic performance in the removal of phenolic pollutants, suggesting its potential in the field of environmental treatment. Future work may be required to further improve the stability and reusability of fungal laccases to develop a powerful biocatalyst for industrial applications.

## Supplementary Information

**Additional file 1: TableS1.** The elemental composition of C and N in pomelo peel and wheat bran (dried mass). **Table S2.** Added amounts of pomelo peel and wheat bran in the fermentation media with different C/N ratios. **Figure S1.** Quantitative analysis of laccase production by *Coriolopsis gallica* CCTCC M 2021731 cultivated in the potato dextrose medium supplemented with guaiacol (0.04%, v/v) in 250 mL shake-flask at 120 rpm and 28 °C for 14 days. **Figure S2. **Chromatogram of *C. gallica* laccase purified by anion exchange. Anion exchange was carried out by DEAE-Sepharose Fast Flow column and gradient elution with citrate/phosphate buffer (pH 6.0–4.0) and NaCl solution (0–1.0 M). **Figure S3. **Chromatogram of *C. gallica* laccase purified by gel filtration. Gel filtration was carried out by Sephadex G-75 column and gradient elution with citrate/phosphate buffer (pH 6.0) and NaCl solution (0–1.0 M).

## Data Availability

The data generated or analyzed during this study are included in this article and its additional materials.
