# Supplementary material for: Green production of a yellow laccase by Coriolopsis gallica for phenolic pollutants removal
Source: AMB Express. 2022 Jul 16;12:96. doi: 10.1186/s13568-022-01434-6 (PMC9288578; doi:10.1186/s13568-022-01434-6)
Supplement: Supplementary file 1 — Additional file 1: TableS1. The elemental composition of C and N in pomelo peel and wheat bran (dried mass). Table S2. Added amounts of pomelo peel and wheat bran in the fermentation media with different C/N ratios. Figure S1. Quantitative analysis of laccase production by Coriolopsis gallica CCTCC M 2021731 cultivated in the potato dextrose medium supplemented with guaiacol (0.04%, v/v) in 250 mL shake-flask at 120 rpm and 28 °C for 14 days. Figure S2. Chromatogram of C. gallica laccase purified by anion exchange. Anion exchange was carried out by DEAE-Sepharose Fast Flow column and gradient elution with citrate/phosphate buffer (pH 6.0–4.0) and NaCl solution (0–1.0 M). Figure S3. Chromatogram of C. gallica laccase purified by gel filtration. Gel filtration was carried out by Sephadex G-75 column and gradient elution with citrate/phosphate buffer (pH 6.0) and NaCl solution (0–1.0 M). [file 13568_2022_1434_MOESM1_ESM.docx]

**Green production of a yellow laccase by *Coriolopsis gallica* for phenolic pollutants removal**

Qingjing Cen^1^, Xiaodan Wu^1,2*^, Leipeng Cao^1^, Yanjuan Lu^3^, Xuan Lu^1^, Jianwen Chen^1^, Guiming Fu^1,2^, Yuhuan Liu^1^, Roger Ruan^4^

^1^State Key Laboratory of Food Science and Technology, Engineering Research Center for Biomass Conversion of Ministry of Education, Nanchang University, Nanchang, Jiangxi 330047, China

^2^International Institute of Food Innovation, Nanchang University, Nanchang, Jiangxi 330047, China

^3^Beijing Fairyland Environmental Technology CO., LTD, Beijing 100096, China

^4^Center for Biorefining, and Department of Bioproducts and Biosystems Engineering, University of Minnesota, St. Paul, MN 55108, USA

^*^Corresponding author: wuxiaodan@ncu.edu.cn

Table S1. The elemental composition of C and N in pomelo peel and wheat bran (dried mass)

| Agricultural residues | Total C (%) | Total N (%) | C/N ratio |
| --- | --- | --- | --- |
| Pomelo peel | 41.35±0.00 | 1.00±0.01 | 41.56 |
| Wheat bran | 40.37±0.01 | 3.30±0.01 | 12.23 |

Table S2. Added amounts of pomelo peel and wheat bran in the fermentation media with different C/N ratios

| C/N ratio | Pomelo peel (g/L) | Wheat bran (g/L) |
| --- | --- | --- |
| 36: 1 | 23.4 | 1.6 |
| 32: 1 | 21.9 | 3.1 |
| 28: 1 | 19.9 | 5.1 |
| 24: 1 | 17.3 | 7.7 |
| 20: 1 | 13.7 | 11.3 |
| 16: 1 | 8.3 | 16.7 |
| 12: 1 | 0 | 25 |





**Fig. S1** Quantitative analysis of laccase production by *Coriolopsis gallica* CCTCC M 2021731 cultivated in the potato dextrose medium supplemented with guaiacol (0.04%, v/v) in 250 mL shake-flask at 120 rpm and 28 ℃ for 14 days.


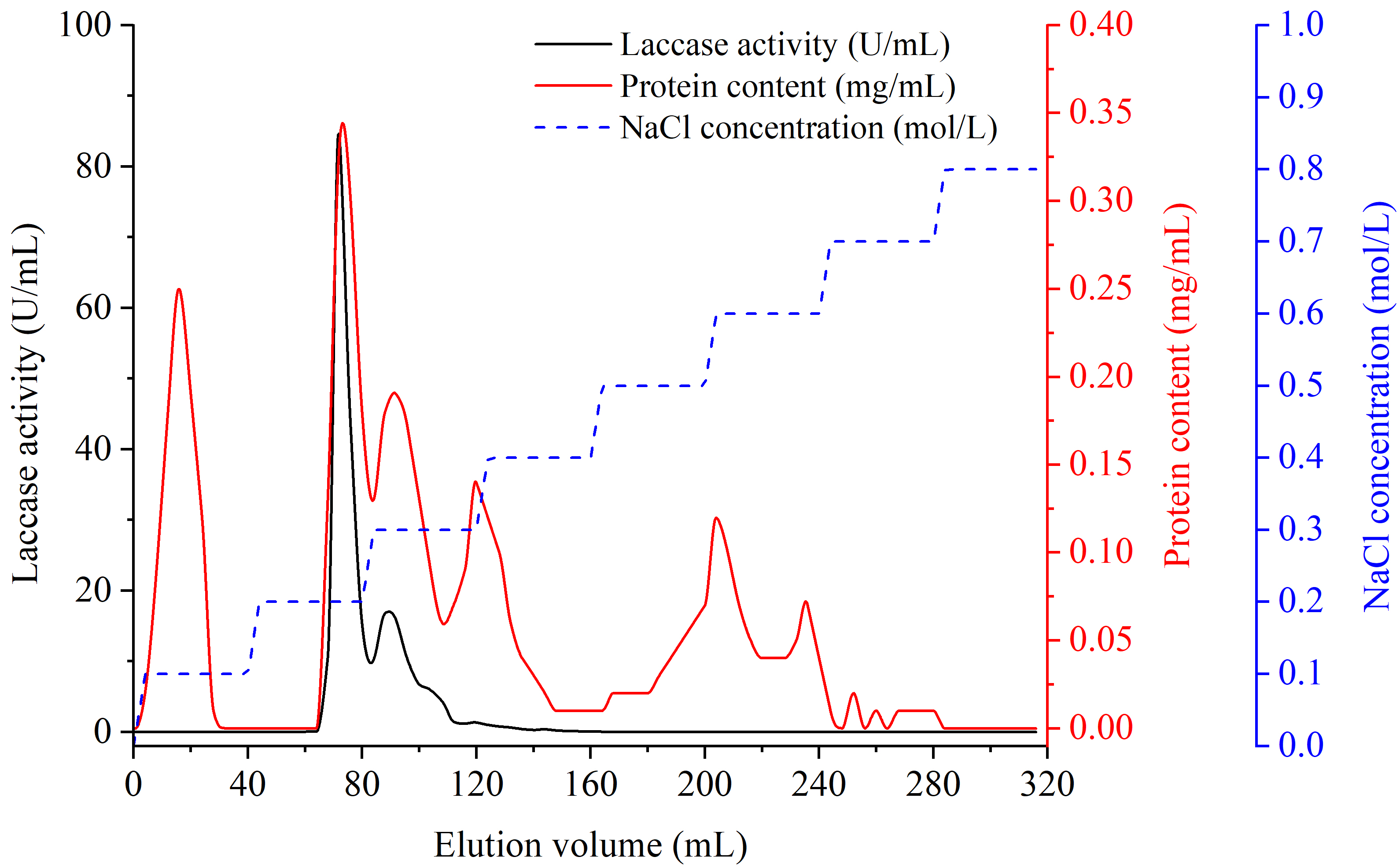


**Fig. S2** Chromatogram of *C. gallica* laccase purified by anion exchange. Anion exchange was carried out by DEAE-Sepharose Fast Flow column and gradient elution with citrate/phosphate buffer (pH 6.0-4.0) and NaCl solution (0-1.0 M).


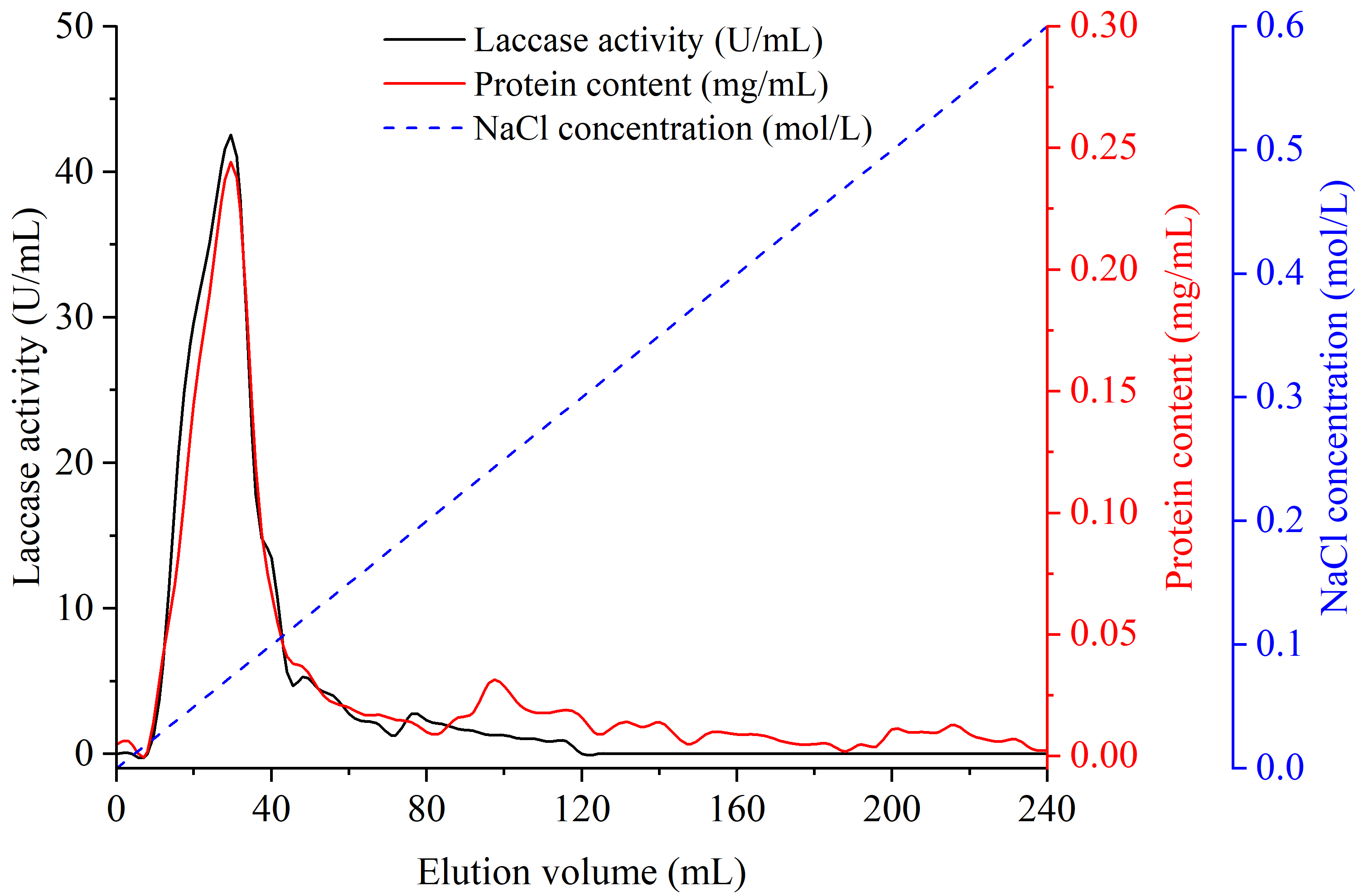


**Fig. S3** Chromatogram of *C. gallica* laccase purified by gel filtration. Gel filtration was carried out by Sephadex G-75 column and gradient elution with citrate/phosphate buffer (pH 6.0) and NaCl solution (0-1.0 M).
